# High-throughput genotype-based population structure analysis of selected buffalo breeds

**DOI:** 10.1093/tas/txab033

**Published:** 2021-05-07

**Authors:** Prakash B Thakor, Ankit T Hinsu, Dhruv R Bhatia, Tejas M Shah, Nilesh Nayee, A Sudhakar, Dharamshibhai N Rank, Chaitanya G Joshi

**Affiliations:** 1 Department of Animal Genetics and Breeding, College of Veterinary Science and Animal Husbandry, Anand Agriculture University, Anand 388001, India; 2 Department of Animal Biotechnology, College of Veterinary Science and Animal Husbandry, Anand Agriculture University, Anand 388001, India; 3 National Dairy Development Board, Anand 388001, India; 4 Gujarat Biotechnology Research Centre, Gandhinagar 382017, India

**Keywords:** admixture, affymetrix, Indian buffalo, linkage disequilibrium, quantitative trait loci, single-nucleotide polymorphism

## Abstract

India is considered as the home tract of some of the best buffalo breeds. However, the genetic structure of the Indian river buffalo is poorly understood. Hence, there is a need to characterize the populations and understand the genetic structure of various buffalo breeds for selection and to design breeding strategies. In this study, we have analyzed genetic variability and population structure of seven buffalo breeds from their respective geographical regions using Axiom Buffalo Genotyping Array. Diversity, as measured by expected heterozygosity, ranged from 0.364 in Surti to 0.384 in Murrah breed, and pair-wise *F*_ST_ values revealed the lowest genetic distance between Murrah and Nili-Ravi (0.0022), while the highest between Surti and Pandharpuri (0.030). Principal component analysis and structure analysis unveiled the differentiation of Surti, Pandharpuri, and Jaffarabadi in first two principal components and at *K* = 4, respectively, while remaining breeds were grouped together as a separate single cluster and admixed. Murrah and Mehsana showed early linkage disequilibrium (LD) decay, while Surti breed showed late decay. In LD blocks to quantitative trait locis (QTLs) concordance analysis, 4.65% of concordance was observed with 873 LD blocks overlapped with 2,330 QTLs. Overall, total 4,090 markers were identified from all LD blocks for six types of traits. Results of this study indicated that these single-nucleotide polymorphism (SNP) markers could differentiate phenotypically distinct breeds like Surti, Pandharpuri, and Jaffarabadi but not others. So, there is a need to develop SNP chip based on SNP markers identified by sequence information of local breeds.

## INTRODUCTION

The importance of genetic diversity in livestock is directly related to the need for genetic improvement of economically important traits as well as to facilitate rapid adaptation to potential changes as per breeding goals. Population structure, and unusual levels of shared ancestry, can potentially cause problems with genome-wide association studies. The analysis of a large number of SNPs across the genome will reveal aspects of the population genetic structure, including evidence of adaptive selection across the genome (Barendse et al., 2009). Domestication of animals have changed the morphological and behavioural characteristics through selection programmes for improving the production traits. That ultimately leads to the formation of very diverse breeds (Diamond, 2002; Toro and Maki-Tanila, 2007; Flori et al., 2009).

River buffalo domestication is likely to have occurred around 6,300 years before present in north-western India (Kumar et al., 2007; Nagarajan et al., 2015). India is one of the largest milk producer in the world, producing over 155.5 million ton milk during 2015–2016 and about 49% of milk production is contributed by buffaloes (FAO, 2007; Department of Animal Husbandry, Fisheries and Dairying, Government of India, 2015). India has approximately 108.7 million buffaloes (Department of Animal Husbandry, Fisheries and Dairying, Government of India, 2015) with 13 registered breeds recognized based on their phenotypic traits, production performance, utility pattern, and eco-geographical distribution.

Genetic analysis is facilitated by genotyping polymorphic genetic loci, also called as genetic variants or markers. SNPs are the most common type of genetic variants, consisting of single-nucleotide differences between two individuals at a particular site in the DNA sequence. Assessing genetic biodiversity and population structure of minor breeds through the information provided by neutral molecular markers, such as SNPs and microsatellites, allows the determination of their extinction risk and to design strategies for their management, conservation, and maintenance of genetic variation for continuous genetic improvement (Toro and Maki-Tanila, 2007). The river buffalo has been selected as a dairy animal with several recognized breeds, spread from the Indian subcontinent to the eastern Mediterranean countries (the Balkans, Italy, and Egypt). For overall breed improvement and to meet future challenges, immediate action is required for the characterization of buffalo breeds in India. Comprehensive knowledge of genetic variation within and among different breeds is necessary for understanding and improving traits of economic importance. Current study was performed based on SNP genotyping data to determine the genetic structure of Indian buffalo breeds so as to construct appropriate conservation strategies and to utilize the breed variation.

## MATERIALS AND METHODS

### Animals and Sampling

A total of 295 female buffaloes from seven breeds were used in this study (Table 1). All animals were selected based on their true breed-specific morphological traits from their respective home tract (Nayee et al., 2016), avoiding sampling from related animals (Supplementary Fig. S1). Blood samples were collected from all the selected animals. This work was ethically approved by the Institutional Animal Ethic Committee (IAEC) of College of Veterinary Sciences and A.H., Anand Agricultural University, Anand (letter no.: IAEC 155/2011).

**Table 1.** Summary of genotyped samples

Sr. no.	Name of breed	Sample origin/breeding tract	Sample size
1	Murrah	Haryana: Rohtak, Hissar, and Jind	70
		Punjab: Nabha and Patiala	
2	Nili-Ravi	Punjab: Amritsar, Gurdaspur, and Ferozepur	33
3	Mehsana	North Gujarat: Mehsana, Banaskantha, and Patan	75
4	Jaffarabadi	Saurashtra, Gujarat: Amreli, Gir, Junagadh, Bhavnagar, and Rajkot	41
5	Banni	Gujarat: Kutchh	20
6	Pandharpuri	Southern Maharashtra: Solapur, Satara, and Latur	34
7	Surti	Southern Gujarat: Anand, Kheda, Baroda, and Surat	22
Total			295

### SNP Genotyping

DNA was extracted using Qiagen QIAamp Blood DNA kit (Qiagen, Germany) as per manufacturer’s instructions. DNA quantity and quality were checked using Nanodrop (Thermo Fisher Scientific, MA) and agarose gel electrophoresis, respectively. SNP genotyping was carried out using commercially available Axiom Buffalo Genotyping Array (90 K) designed with 123,040 probes on Gene Titan MC (Thermo Fisher Scientific, MA) instrument at a commercial laboratory (Imperial Life Science Group, Gurgaon). It was designed based on SNPs discovered from Mediterranean, Murrah, Jaffarabadi, and Nili-Ravi breeds of buffaloes. In the designed array, there are 123,040 probes, which include 89,988 probes for SNPs, while other probes are for quality control (QC) and gender calling.

### Data Filtering and Quality Control

Only SNPs mapped to autosomal chromosomes were used in this study. Data were filtered with PLINK v1.07 (Purcell et al., 2007) based on criteria: removal of SNPs with same UMD position, missing genotypes (<0.1), minor allele frequency (<0.05) and Hardy–Weinberg Equilibrium (*P*-value <0.00001; Supplementary Table S1). None of the animal/individual was removed during the quality filtering, whereas 75,704 SNPs remained after filtering.

### Genetic Diversity Assessment

Observed and expected genotype frequencies within each breed was calculated for all the loci using PLINK v1.07 and the results were evaluated based on *P*-values for significance test for Hardy-Weinberg Equilibrium, obtained for each loci. Linkage disequilibrium (LD) was calculated using PLINK and *r*^2^ values were calculated for all SNP pairs that were located less than 1,000 SNPs apart and falling under 10 Mb distance windows. Furthermore, SNPs were binned with bin size of 10,000 bases distance, and average *r*^2^ value of each bin was plotted against median distance value ggplot2 v2.2.1 package in R v3.3. Pair-wise *F*_ST_ values and the associated 95% confidence intervals were calculated using the Hierfstat package (Goudet, 2005) in R. Wright’s inbreeding coefficient estimated as *F*_IS_, which is caused by Wahlund effect by mixing individuals from genetically different populations, and normalized variance in allele frequencies between populations is estimated as *F*_ST_ (Zhivotovsky, 2015). Pair-wise *F*_ST_ values between all possible combinations of breeds were estimated and subsequently phylogenetic tree was generated in Fitch–Phylip using Fitch–Margoliash method, which uses a weighted least squares method for clustering based on genetic distance (Fitch and Margoliash, 1967).

Several statistical parameters were stated to measure the extent of LD. The *r*^2^ is a better descriptor of LD as it is more robust and not sensitive to changing gene frequency and effective population size (Zhao et al., 2007). Effective population size can be estimated for several past generations for given population using the available information of correlation between gene frequencies and LD (Sved, 1971). The decline rate of LD with intermarker distance was estimated using bin size of 10 kb distance between SNPs.

Breed-wise effective population size (Ne) was calculated using SNeP v1.1 (Barbato et al., 2015) with parameters: bin-width = 50,000 bp; minimum distance between SNPs = 50,000 bp, maximum distance between SNPs = 4,000,000 bp and minimum allele frequency = 0.05. SNeP estimates Ne from genome-wide LD using the method suggested by Corbin et al. (2012). Population clustering was performed using principal component analysis in order to place the breed groups with respect to their genetic constitutes with PLINK-1.9 (Chang et al., 2015) using 285 highly variable markers (allele frequency difference between breeds >0.5) and plotted using scatterplot3d package in R. Breed structure and breed differentiation was performed using fastSTRUCTURE (Raj et al., 2014) using same 285 highly variable markers. The differentiation of populations was performed up to the group (K) level of 8 using simple model. The fastSTRUCTURE analysis provided ancestry proportions for each sample under analysis, which was graphically represented by distruct.py script within the fastSTRUCTURE software.

### Genome-Wide LD Block Mapping on Quantitative Trait Locis (QTLs)

Linkage disequilibrium blocks, combination of alleles linked along a chromosome and inherited together from a common ancestor, were generated with Java-based gPLINK v1.0 and Haploview v2.01 (Barrett et al., 2004). Blocks were defined by employing haplotypic diversity criterion, where a small number of common haplotypes provide high chromosomal frequency coverage (Patil et al., 2001; Zhang et al., 2002, 2003; Anderson and Novembre, 2003). The algorithm suggested by Gabriel et al. (2002) was used, which defines a pair of SNPs to be in strong LD if the upper 95% confidence bound of D′ value is between 0.7 and 0.98. Reconstructed haplotypes were inserted into Haploview v2.01 to estimate LD statistics and construct the blocking pattern for all 29 autosomes. LD blocks were estimated using an accelerated Expectation–Maximization algorithm method described by Qin et al. (2002). QTL database was retrieved from previously reported QTLs in Animal QTLdb (Hu et al., 2013). QTL data set of cattle (*Bos taurus*) QTL_UMD_3.1.1 was used as a reference for the analysis, containing the information regarding six types of the traits: milk traits; health traits; production traits; reproduction traits; exterior traits; and meat and carcass traits. The QTL files were intersected with the files of LD blocks using Bedtools v2.26.0 (Quinlan and Hall, 2010) to obtain information of QTLs overlapping with LD blocks.

## RESULTS

### Genetic Diversity Analysis

Samples were genotyped with the average call rate of passed sample 98.58%. Upon applying QC measures, 295 samples and 75,704 SNPs remained for population analysis (Supplementary Table S1).

### Allele Frequency-Based Differentiation

**Figure 1.**
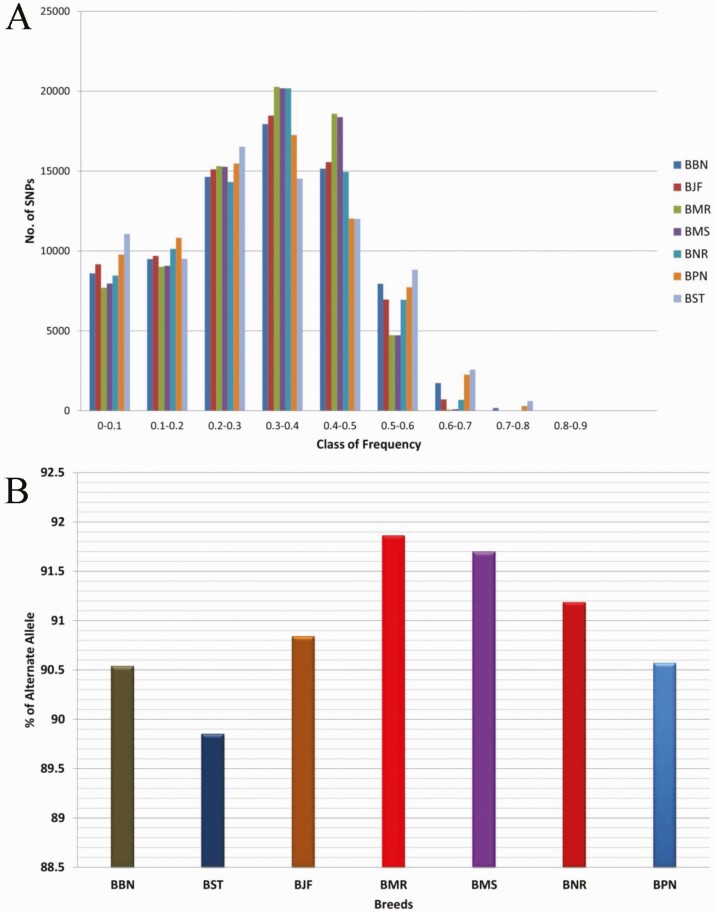
Alternate allele distribution. (A) Distribution of alternate allele frequency in studied buffalo breed. (B) Breed-wise proportion and distribution of alternate allele with allele frequency >0 (monomorphic SNPs were removed; BBN: Banni, BJF: Jaffarabadi, BMR: Murrah, BNR: Nili-Ravi, BMS: Mehsana, BPN: Pandharpuri, BST: Surti).

Highest number of SNPs with alternate allele frequency between 0.3 and 0.4 was observed in all studied buffalo breeds except Surti (Fig. 1A). Highest allele count was observed in the range of frequency class 0.2–0.5. Highest average alternate allele frequency was observed in Nili-Ravi (0.3051), while Jaffarabadi showed least (0.3028) among all breeds (Supplementary Fig. S2). The distribution of alternate allele did not significantly differ between studied breeds. Highest proportion of alternate alleles was observed in Murrah with 91.86%, while lowest proportion was observed in Surti with 89.86% (Fig. 1B). The observed heterozygosity (*H*_*O*_) and expected heterozygosity (*H*_*E*_) in all breeds did not differ and ranged from 0.3719 (Pandharpuri) to 0.3864 (Murrah) and 0.3643 (Surti) to 0.3846 (Murrah), respectively (Table 2). The lowest *F*_IS_ was observed for Murrah (−0.0046) followed by Mehsana (−0.0070), while comparative higher values were observed in Surti (−0.0314) followed by Banni (−0.0270).

**Table 2.** Genetic diversity parameters in Indian buffalo breeds from genotyped data (BBN: Banni, BJF: Jaffarabadi, BMR: Murrah, BNR: Nili-Ravi, BMS: Mehsana, BPN: Pandharpuri, BST: Surti)

Breed	Number of Animals	Observed heterozygosity, *H*_*O*_ (mean ± SE)	Expected heterozygosity, *H*_*E*_ (mean ± SE)	Inbreeding coefficient, *F*_IS_ (mean ± SE)
BBN	20	0.3839 ± 0.0006	0.3738 ± 0.0005	−0.0270 ± 0.0036
BMS	75	0.3857 ± 0.0005	0.3830 ± 0.0005	−0.0070 ± 0.0033
BNR	33	0.3832 ± 0.0006	0.3799 ± 0.0005	−0.0089 ± 0.0072
BPN	34	0.3719 ± 0.0006	0.3680 ± 0.0005	−0.0107 ± 0.0116
BJF	41	0.3839 ± 0.0006	0.3738 ± 0.0005	−0.0098 ± 0.0031
BMR	70	0.3864 ± 0.0005	0.3846 ± 0.0005	−0.0046 ± 0.0024
BST	22	0.3757 ± 0.0007	0.3643 ± 0.0005	−0.0314 ± 0.0094

### *F*_ST_-Based Differentiation

**Figure 2.**
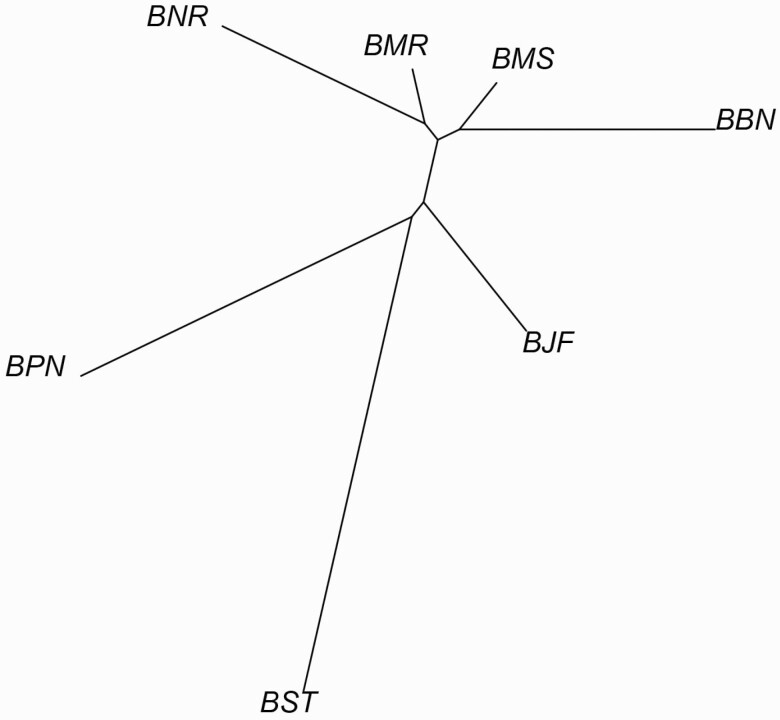
Phylogenetic tree of breed differentiation based on pair-wise *F*_ST_ values. Labeled tree with the name of breed at each leaf (BBN: Banni, BJF: Jaffarabadi, BMR: Murrah, BNR: Nili-Ravi, BMS: Mehsana, BPN: Pandharpuri, BST: Surti).

*F*_*ST*_ values showed least genetic distance between Murrah and Nili-Ravi (0.00221) followed by Murrah and Mehsana (0.00402), while highest genetic distance was observed between Surti and Pandharpuri (0.03097) followed by Surti and Banni (0.02650; Supplementary Table S2). Based on *F*_*ST*_ values, neighbor-joining tree placed Nili-Ravi and Murrah, as well as Mehsana and Banni together in two separate clusters, which corresponds with their geographical origin (Fig. 2). Furthermore, this clustering pattern was also supported by neighbor-joining tree generated using studied SNPs (Supplementary Fig. S3). This differentiation also correlates with the morphological differentiation of the buffalo breeds.

### Principal Component Analysis (PCA) Results

**Figure 3.**
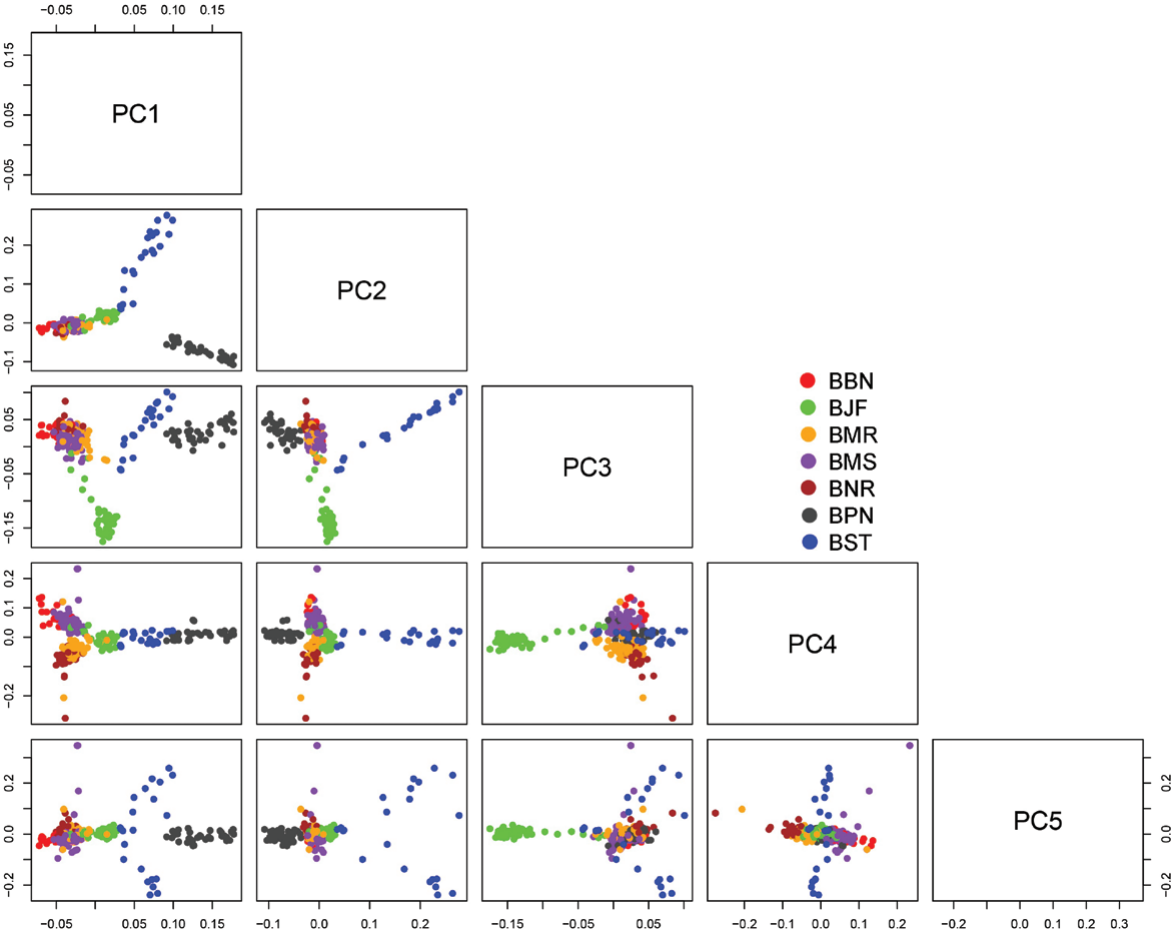
2D principal component analysis (PCA) plot of all seven buffalo breeds together up to principal components 5 (BBN: Banni, BJF: Jaffarabadi, BMR: Murrah, BNR: Nili-Ravi, BMS: Mehsana, BPN: Pandharpuri, BST: Surti).

The total variability explained by first three principal components was 65.6%, of which first, second, and third components explained 30.05%, 27.14%, and 8.45%, respectively. This variation resulted in a separate cluster of Surti, Pandharpuri, and Jaffarabadi on coordinates 1, 2, and 3, respectively, while other breeds remain admixed (Fig. 3).

### Model-Based Population Assignment

**Figure 4.**
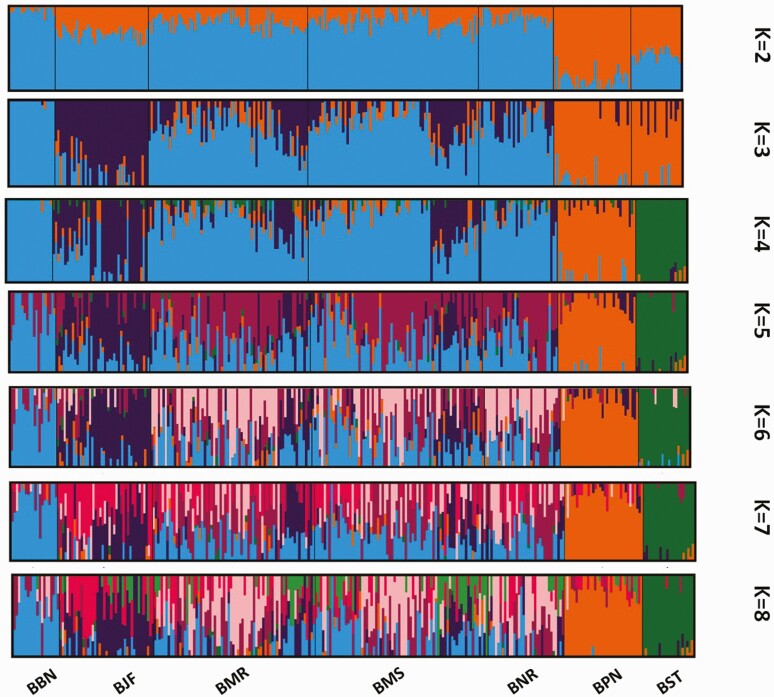
Estimated population structure by fastSTRUCTURE for *K* = 2 to *K* = 8. Each individual is represented by a thin vertical line, and each breed is demarcated by a thick vertical black line (BBN: Banni, BJF: Jaffarabadi, BMR: Murrah, BNR: Nili-Ravi, BMS: Mehsana, BPN: Pandharpuri, BST: Surti).

Furthermore, relatedness between breeds and the significance of the existence of subpopulations was investigated by model-based unsupervised clustering using *K* = 2 to *K* = 8 (*K* values indicates the number of groups). For K = 3, total three breed groups were identified (Fig. 4); cluster I included Banni, Murrah, Mehsana, and Nili-Ravi breeds; cluster II included Jaffarabadi breed only; and cluster III grouped Pandharpuri and Surti breeds. But when K = 4 was assumed, cluster III further split into independent cluster for Surti breed. These clusters obtained were consistent with the neighbor-joining tree. The membership of Cluster I was consistent with breed histories, with one cluster including a pair of closely related breeds (Murrah and Mehsana), which showed some level of admixture. It seemed to be an optimum of four clusters, which was also indicated by a maximum likelihood method (Supplementary Fig. S4). So, *K* = 4 was considered to represent most relevant number of genetic clusters in the data sets, which corresponded to their breed designation. Surti breed showed better separation with small amount of admixture at all levels, while Murrah and Mehsana breed showed higher amount of admixture consistent with its crossing with other breeds. With increasing *K* values, Pandharpuri and Surti showed separation at all subsequent levels (Fig. 4). Three Jaffarabadi individuals were identified as pure breed based on *Q*-value greater than 95%, while the remaining showed variable amount of admixture. Similarly, Pandharpuri buffaloes showed the highest number (26) of purebred individuals with more than 80% of *Q*-value. Likewise, Surti breed has 19 purebred individuals with negligible admixture with other breeds.

### LD Analysis

**Figure 5.**
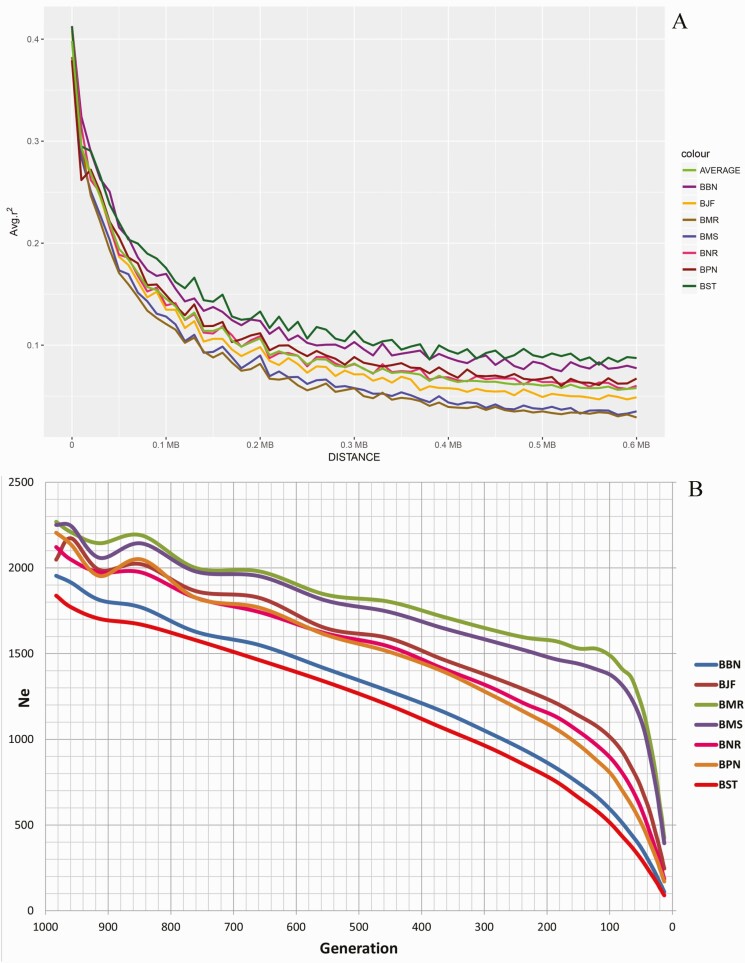
LD study of Buffalo breeds: (A) LD decay plot based on all pair-wise comparisons between adjacent loci of all seven breeds. The horizontal axis depicts the intermarker distance in base pair and vertical axis shows the average *r*^2^ values. (B) Effective population size (Ne) of different breeds with respect to generation time (BBN: Banni, BJF: Jaffarabadi, BMR: Murrah, BNR: Nili-Ravi, BMS: Mehsana, BPN: Pandharpuri, BST: Surti).

LD decay showed highest *r*^2^ value in Surti (from 0.412 to 0.175) followed by Banni (from 0.412 to 0.169; Fig. 5A), while Pandharpuri (from 0.379 to 0.149) and Nili-Ravi (from 0.412 to 0.139), as well as Mehsana (from 0.378 to 0.128) and Murrah (from 0.382 to 0.120), decayed almost with the same rate. In Surti breed, LD decayed late as distance between loci increased compared to other breeds. However, Mehsana and Murrah showed early decay among all the breeds.

A continuous steady decline in effective population size was observed over the last 1,000 generations in all breeds. Effective population size of Murrah and Mehsana has drastically declined over the last 100 generations with steeper slope, while Surti and Banni are declining at lower rate (Fig. 5B). Jaffarabadi, Nili-Ravi, and Pandharpuri showed intermediate rate of declination over the last 100 generations.

### Genome-Wide Study of LD blocks

**Figure 6.**
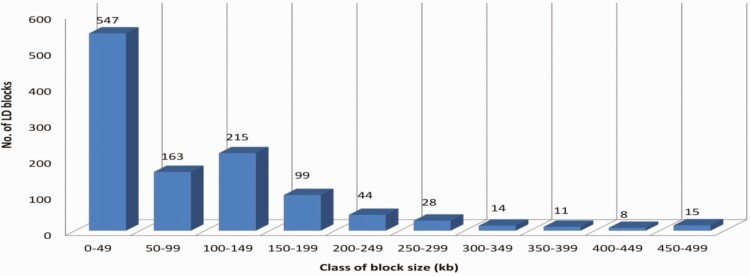
LD blocks distribution based on the size of block in respective class of size (in kb).

Total 1,144 LD blocks were obtained with the highest number of blocks on chromosome 1 (99 blocks), while the least number of blocks on chromosome 28 with 19 blocks (Table 3). Overall, the mean number of SNPs in block ranged from 2.75 to 4.54 SNPs per chromosome, while the maximum number of SNPs per block ranged from 5 (chromosome 18) to 16 (chromosome 17). Overall, frequency-based size distribution of LD blocks revealed that the highest number (547) of LD blocks were found having sizes less than 50 kb, while very few (15) were observed having sizes as high as 450–499 kb (Fig. 6).

**Table 3.** Chromosome-wise LD block distribution statistics with total number of LD blocks, average block size, mean, and maximum number of SNPs in blocks

Chromosome	Total LD blocks	Mean number of SNPs per block	Max. number of SNPs in blocks
1	99	3.48	7
2	87	3.68	9
3	59	3.25	6
4	58	3.44	8
5	63	3.73	15
6	43	3.72	9
7	44	3.72	15
8	52	3.75	10
9	39	4.00	8
10	36	3.94	6
11	54	3.51	9
12	37	3.75	9
13	38	3.34	9
14	31	2.93	13
15	33	3.00	6
16	44	3.56	12
17	30	3.83	16
18	24	3.04	5
19	31	4.54	11
20	23	3.47	9
21	36	3.94	11
22	26	3.76	13
23	22	3.72	7
24	27	2.96	7
25	29	2.75	9
26	16	3.56	8
27	23	3.34	8
28	19	3.84	7
29	22	3.77	10
All	1,145	3.56	

#### LD blocks—QTL concordance.

Out of 1,144 LD blocks (4,090 markers), 436 (1,624 markers), 368 (1,285 markers), 326 (1,253 markers), 345 (1,351 markers), 104 (426 markers), and 81 LD blocks (338 markers) overlapped with QTLs for traits, such as milk production (Supplementary Fig. S5), production (Supplementary Fig. S6), reproduction (Supplementary Fig. S7), meat and carcass (Supplementary Fig. S8), health (Supplementary Fig. S9), and exterior (Supplementary Fig. S10), respectively. Concordance, measured as a proportion of LD blocks and QTLs overlapping each other, was highest on chromosome 1 (16.91%) and lowest on chromosome 14 (0.91%). Overall, the concordance of all the chromosomes together was 4.65%, with 873 LD blocks intersected with 2,330 QTLs (Table 4). Chromosome-wise distribution of LD blocks, number of markers, and mapped QTLs for respective traits is shown in Supplementary Table S3.

**Table 4.** Chromosome-wise distribution of LD blocks and QTLs with its percentage of concordance and discordance

Chromosome	No. of QTLs	No. of QTLs overlapped by LD blocks	No. of LD blocks	No. of LD blocks overlapped with QTLs	Concordance between QTL and LD blocks in %
1	2,403	325	99	98	16.91
2	2,711	163	87	56	7.83
3	2,780	55	58	43	3.45
4	4,440	31	58	21	1.16
5	3,534	103	63	56	4.42
6	10,483	237	43	41	2.64
7	2,089	63	44	41	4.88
8	1,177	55	52	45	8.14
9	1,289	61	39	21	6.17
10	1,839	78	36	26	5.55
11	3,163	118	54	34	4.72
12	1,046	60	37	26	7.94
13	1,775	101	38	25	6.95
14	7,293	38	31	29	0.91
15	1,050	32	33	32	5.91
16	1,236	63	44	37	7.81
17	1,548	47	30	26	4.63
18	1,233	27	24	21	3.82
19	1,735	73	31	18	5.15
20	2,914	140	23	21	5.48
21	1,184	56	36	23	6.48
22	946	38	26	17	5.66
23	1,004	120	22	21	13.74
24	754	11	27	12	2.94
25	1,802	101	29	25	6.88
26	3,856	52	16	16	1.78
27	747	27	23	19	5.97
28	643	27	19	16	6.50
29	1,130	28	22	17	3.91
Combined	67,804	2,330	1,144	873	4.65

Furthermore, analysis was also performed based on markers overlapping with QTLs of milk fat percentage (143 markers) and body weight (315 markers) using phenotypic recorded data from National Dairy Development Board (India) (www.nddb.coop) and Central Institute for Research on Buffalo (India), (cirb.res.in) respectively. Surprisingly, no particular pattern was observed linking phenotypic data (literature-based QTLs of milk fat and body weight) with trait-specific marker-based separation.

## DISCUSSION

Genetic diversity studies conducted for buffalo in India have previously relied primarily on the use of microsatellites markers (Pundir et al., 2000; Kumar et al., 2006; Tantia et al., 2006; Kataria et al., 2009; Joshi et al., 2013; Joshi et al., 2015), while the use of SNP genotype data in Indian cattle has also been previously reported (Dash et al., 2017). Previously, Perez-Pardal et al. (2018) have performed the study on 15 buffalo animals each from river and swamp buffalo using cattle SNP array (Illumina BovineHD BeadChip) and they have confirmed that analysis has better suitability for population structure, hybridization, and breed identification of water buffalo populations.

The chip used in this study was designed based on SNP markers of four breeds (Mediterranean, Murrah, Nili-Ravi, and Jaffarabadi) although using the reference of *Bos taurus* (UMD_3.1 assembly) (Iamartino et al., 2013). The differences in allele frequencies among the breeds may be caused by genetic drift, adaptation to selection, or ancient divergence among founder populations (MacEachern et al., 2009; Dadi et al., 2012). Therefore, these SNPs identified in this study will be useful for the study on breed structure identification and population differentiation. Here, we used the term “alternate allele” in place of “minor allele” because minor allele frequency does not exceed over 0.5, while, in this study, the allele frequency exceeds over 0.5, often called as “fixed allele,” and, hence, it has been considered as an “alternate allele.” The differences in observed allele frequencies among breeds show the genetic diversity that exists within and between the breeds. Murrah and Mehsana had the highest number of SNPs with intermediate class of frequency, suggesting that this array could be utilized for these breeds for association studies. The higher genetic variability observed in the Murrah and Mehsana, which is evident from the population structure analysis, suggests the introgression of these breeds with other breeds, such as Banni, Nili-Ravi, and Jaffarabadi, while Surti and Pandharpuri showed less polymorphic SNPs, suggesting less genetic variability. These findings are further supported by *H*_*O*_ and *H*_*E*_ values, which were found to be higher in Murrah and Mehsana breeds as compared to other breeds, which could be due to the availability of large population of these breeds owing to their higher milk production potential, whereas other breeds are limited in numbers. Pandharpuri and Surti showed less genetic variability with the lowest HE suggesting that inbreeding in conjunction with a small population size and resulted in a loss of variation within the breed. This type of low diversity among breeds was previously reported in other studies of cattle and buffalo using microsatellites (Machado et al., 2003; Sraphet et al., 2008; Suh et al., 2014) and using SNP panels (Dash et al., 2017).

In this study, the mean *F*_ST_ indicated that a pair of Surti and Pandharpuri population has greater genetic distance than other pairs. Pair-wise *F*_ST_ between these buffalo breeds was significantly different from zero (*P* < 0.05). Genetic differentiation (pair-wise *F*_ST_) indices observed in the present study are sufficient to explain the fact that these buffalo breeds are geographically well separated from each other, and we had reported a similar observation in our previous study among Western-Central Indian cattle breeds (Shah et al., 2013). Our *F*_ST_-based genetic classification was in agreement with this classification of buffaloes except the separation of Jaffarabadi breed. However, the results failed to explain the hypothesis that Mehsana breed has been developed using Murrah bulls on local Surti buffaloes (Pundir et al., 2000) as both the breeds clustered separately. Earlier study based on microsatellite markers revealed genetic diversity (*F*_ST_) based clustering between Mehsana with Jaffarabadi and Surti with Pandharpuri (Kumar et al., 2007). Our study also showed clustering among Surti and Pandharpuri, while Mehsana and Jaffarabadi formed separate clusters.

The results of the PCA analysis revealed the higher amount of genetic similarities among Murrah, Mehsana, Banni, and Nili-Ravi, while Surti, Jaffarabadi, and Pandharpuri showed greater genetic differentiations with three distinct clusters. The clustering of populations from both the PCA and fastSTRUCTURE indicated low levels of within-population diversity of the Surti, Jaffarabadi, and Pandharpuri breeds and higher divergences of these populations from the Murrah, Mehsana, Banni, and Nili-Ravi breeds. In the current study, Surti, Jaffarabadi, and Pandharpuri grouped in separate clusters, contrary to earlier microsatellite-based study, where all these breeds clustered together (Kumar et al., 2006). The high genetic diversity and distinct breed structure imply the possibility of selective breeding in these Indian buffalo breeds for genetic improvement (Murrah and Mehsana). Four breeds (Surti, Pandharpuri, Jaffarabadi and Banni) were distinctly separated while two breeds (Murrah and Mehsana) showed more admixtures. Admixture was detected in Cluster I of the ancestral clusters, whereas the breeds within remaining clusters were more differentiated. High admixture was observed between Murrah and Mehsana breed, reflecting crossbreeding between these breeds. The probable reason for observed admixture in Mehsana could be an outcome of gene flow from Murrah males in the recent past (Kumar et al., 2006) or they might be the same breed, which was domesticated in different geographical regions. Kumar et al. (2006) evaluated the breed admixture using microsatellite markers, and results revealed that the three different clusters contributed mainly from the Toda, Jaffarabadi, and Pandharpuri animals, with a very high membership coefficient. The research also stated that there was an anecdotal evidence to indicate that the Mehsana breed has been an outcome of gene flow from the Murrah males in the recent past. Nili-Ravi and Murrah have higher average allele frequencies, which can be due to biasness to SNP selection from both Nili-Ravi and Murrah as reference during SNP chip designing.

LD decay used to study the linkage of markers with increase in intermarker distance and was used to decide appropriate intermarker distance for different populations. The magnitude of LD and its decay with genetic distance determine the resolution of association mapping and are useful for assessing the desired numbers of SNPs on arrays. The results of LD decay illustrate Surti breed showing early decay as compared to other breeds, while Mehsana and Murrah breeds showed late decay together, which could be assumed as they are under strong selection pressure. Similar results were obtained by Dash et al. (2017) using HD SNP chip for Indian cattle breeds where Sahiwal and Tharparkar breeds showed late decay. These results reflected that the Surti breed has smaller population size as it got decayed earlier. Other breeds also exhibited LD decay as per their available breedable population. Larger the population size, longer the LD decay. Effective population size of Murrah and Mehsana has drastically declined over the last 100 generations. The probable cause of drastic decline in Ne for Murrah and Mehsana may be attributed to selection efforts done by traditional farmers, as well as the use of AI in the native tract of these breeds. It is believed that Mehsana breed has been developed a couple of decades ago from Murrah and Surti buffalo (might have completed less than 100 generations). Hence, the results should be viewed considering theoretical expectations. It gives information regarding effective population size of ancestors. Shin et al. (2013) estimated the effective population size in Korean cattle using HD SNP chip, which revealed rapid increase in effective population size over the past 10 generations with the values increasing 5-fold (close to 500) by 10 generations. Santana et al. (2011) also reported a small effective size of 40 from several Murrah herds based on phenotypic recordings and average relatedness. An effective population size of at least 50 animals is enough to prevent inbreeding depression, the minimum level recommended by the Food and Agriculture Organisation of the United Nations. The *H*_*O*_ level is similar in all breeds studied irrespective of their population size, but still the present results should be interpreted with caution as, for some breeds, less than 50 animals were tested.

The haplotype block structure and its distribution in the genome of cattle, especially studies based on high-density SNPs, have been rarely reported (Villa-Angulo et al., 2009). However, Bohmanova et al. (2010) have performed study on LD for identifying the genomic region in American Holstein and they have concluded that LD values get inflated by a small population and strongly depend on allele frequency. Thus, the current analysis was performed to construct the haplotype structure in the buffalo genome and to detect the relevant genes affecting quantitative traits. Jiang et al. (2010) identified the milk trait QTL-specific SNPs in cattle and found that a large proportion of the significant SNPs (61 out of 105) were located on BTA14 and also within the reported QTL regions. In our study, 76 QTLs (mostly of milk protein percentage, milk yield, and milk fat percentage) on chromosome 20 were concordant with 13 LD blocks. Mai et al. (2010) recognized total 98 QTLs for milk production trait, which included 30 for milk index, 50 for fat index, and 18 for protein index. The density of QTLs of body weight was higher on chromosome 23 along with other productive traits. Mai et al. (2010) also reported a greater number of significant SNP associations for production (54) than for fertility traits (29) with 22 QTL regions associated with fertility traits and 14 with production traits. Li et al. (2018) have used 90K Affymetrix Axiom Buffalo SNP Array to identify the SNPs, genomic regions, and genes that were associated with reproductive traits, and they have found a total of 40 suggestive loci (related to 28 genes) that were identified to be associated with six reproductive traits (first, second, and third calving age, calving interval, the number of services per conception, and open days). The concordance study of meat and carcass trait revealed that the largest QTL of shear force was observed on chromosome 6 and QTL of tridecylic acid content located on chromosome 15. Wu et al. (2014) studied the carcass trait of Simmental cattle and identified that the genes in the beef cattle genome significantly associated with foreshank weight and triglyceride levels. A total of 12 and 7 SNPs in the bovine genome were significantly associated with foreshank weight and triglyceride levels, respectively.

In the concordance analysis of exterior traits, majorly the QTLs were associated with udder traits (udder swelling score QTL, udder depth QTL, udder attachment QTL, teat length QTL, etc.). This information of genotypes could be used to associate phenotypes and perform the selection. Based on the above results, we can assume that exterior traits are less important for the association of QTL with LD block or haplotypes due to the insufficient size of QTL and low proportion of concordant QTL with LD blocks. van den Berg et al. (2014) studied the concordance for a leg conformation trait in dairy cattle and QTL status was used in a concordance analysis to reduce the number of candidate mutations. In the concordance study of health trait, QTLs associated with somatic cell count were observed almost on every chromosome. The larger-size QTL of cold tolerance was observed on chromosome 7. More numbers of QTLs associated with bovine tuberculosis susceptibility were found on chromosome 20 and QTLs for clinical mastitis found on chromosome 14 as well as on chromosome 24. Raphaka et al. (2017) identified the markers associated with tuberculosis on *Bos taurus* autosomes (BTA) 2 and on BTA 23 and concluded a major role of BTA 23 for susceptibility to bovine tuberculosis.

## CONCLUSION

The study of population structure analysis in Indian buffaloes based on SNPs revealed that the distribution of SNP markers across the buffalo genome of all breeds studied was almost similar. Genetic parameters, such as *H*_*E*_ and *H*_*O*_, showed the absence of genetic differentiation while *F*_IS_ was able to differentiate Banni and Surti animals. Furthermore, *F*_ST_ was able to differentiate the buffaloes as per their respective geographical locations. The levels of SNPs variation in this study could be insufficient to differentiate the other local breed except Pandharpuri, Surti, and Jaffarabadi (morphological distinct breeds), so there is a need to develop SNP chip based on SNP markers identified by sequence information of local buffalo breeds. LD block QTLs concordance study could explore a new window for genomic selection in animals and, therefore, the development of new SNP chip based on information of buffalo genome and buffalo-specific genetic technologies is warranted.

## Supplementary Material

txab033_suppl_Supplementary_Figures

txab033_suppl_Supplementary_Tables

## Data Availability

The data sets generated and/or analyzed during the current study are not publicly available [Intellectual Property Rights likely to be generated out of this and hence the data are not provided publically] but are available from the corresponding author on reasonable request.
