# Supplementary figures and images for: High-throughput genotype-based population structure analysis of selected buffalo breeds
Source: Transl Anim Sci. 2021 May 7;5(2):txab033. doi: 10.1093/tas/txab033 (PMC8103726; doi:10.1093/tas/txab033)

Fig S1:

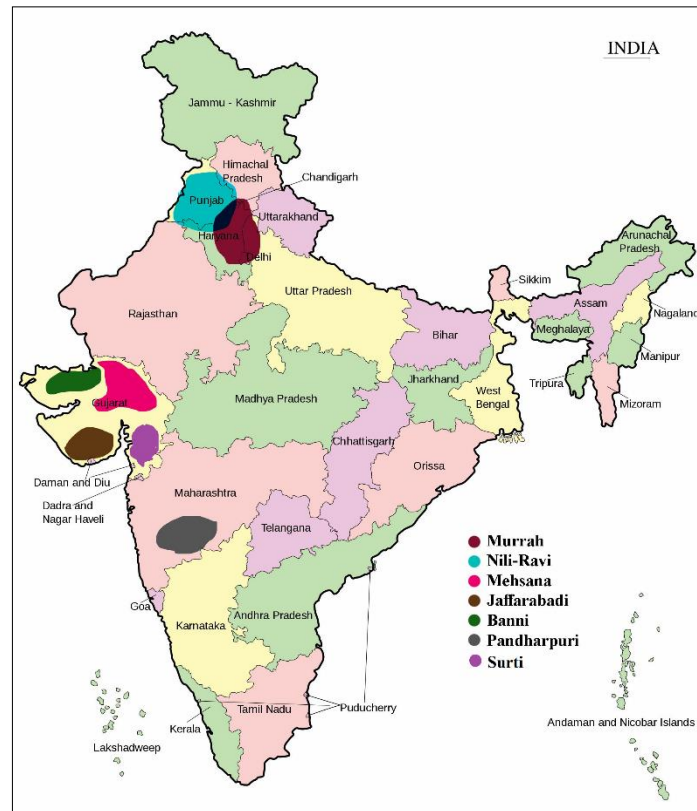

Fig S2:

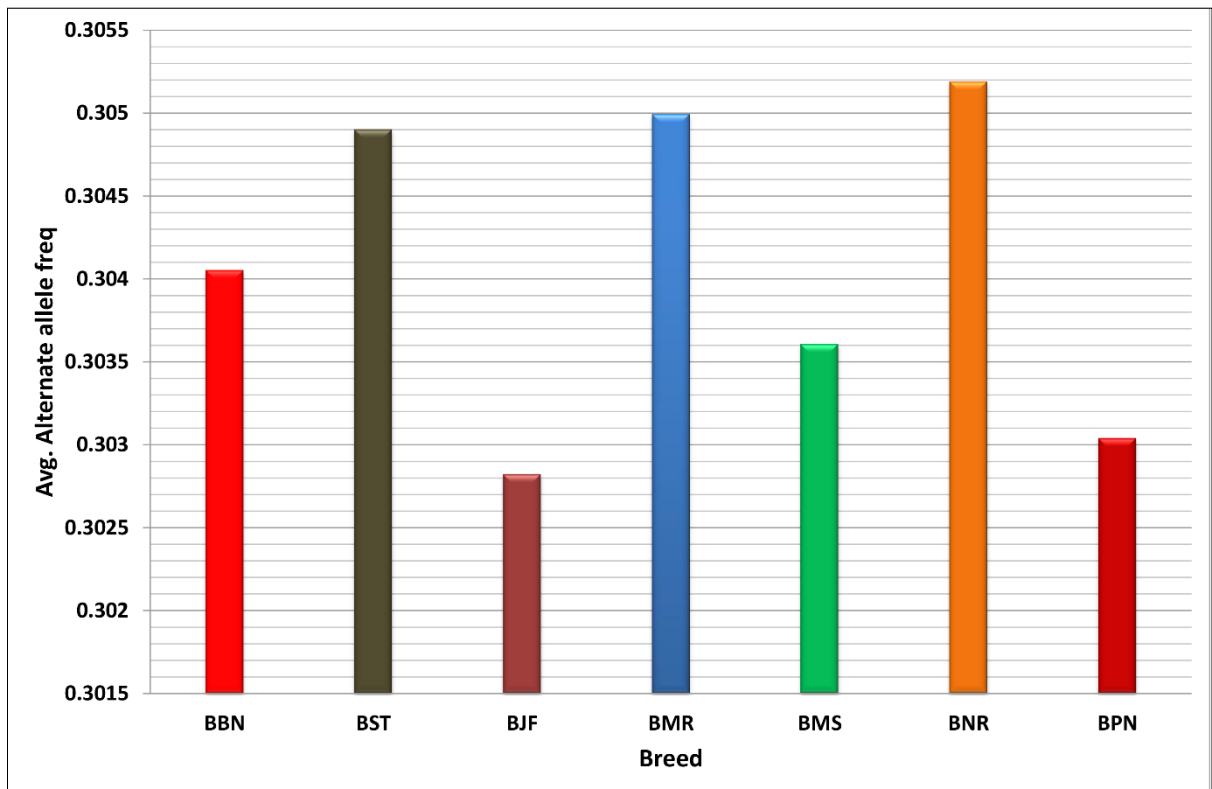

**Fig S3:**

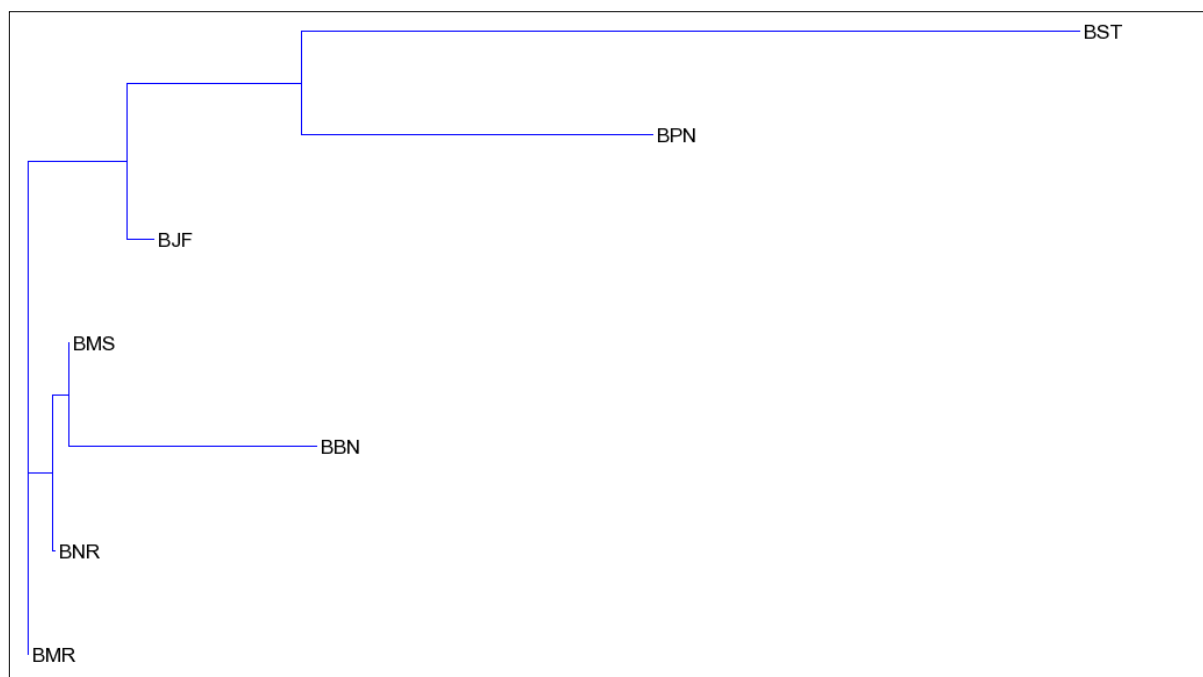

**Fig S4:**

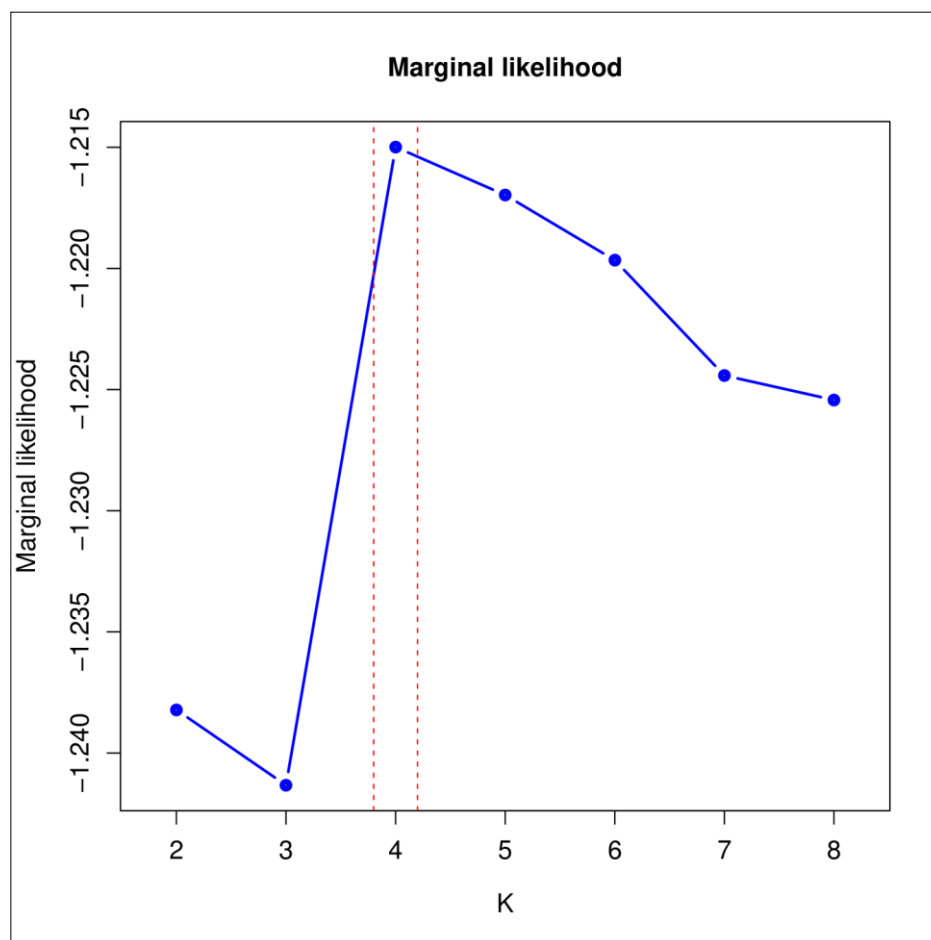

Fig S5:

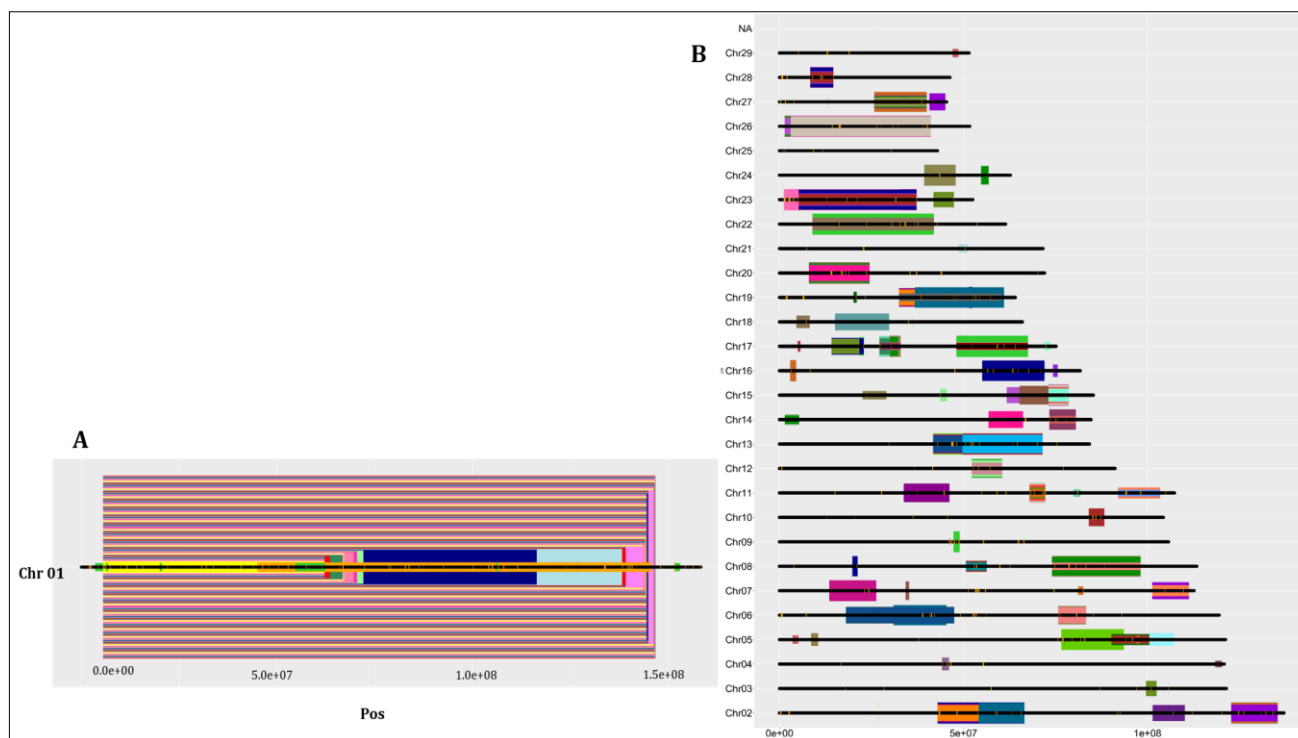

Fig S6:

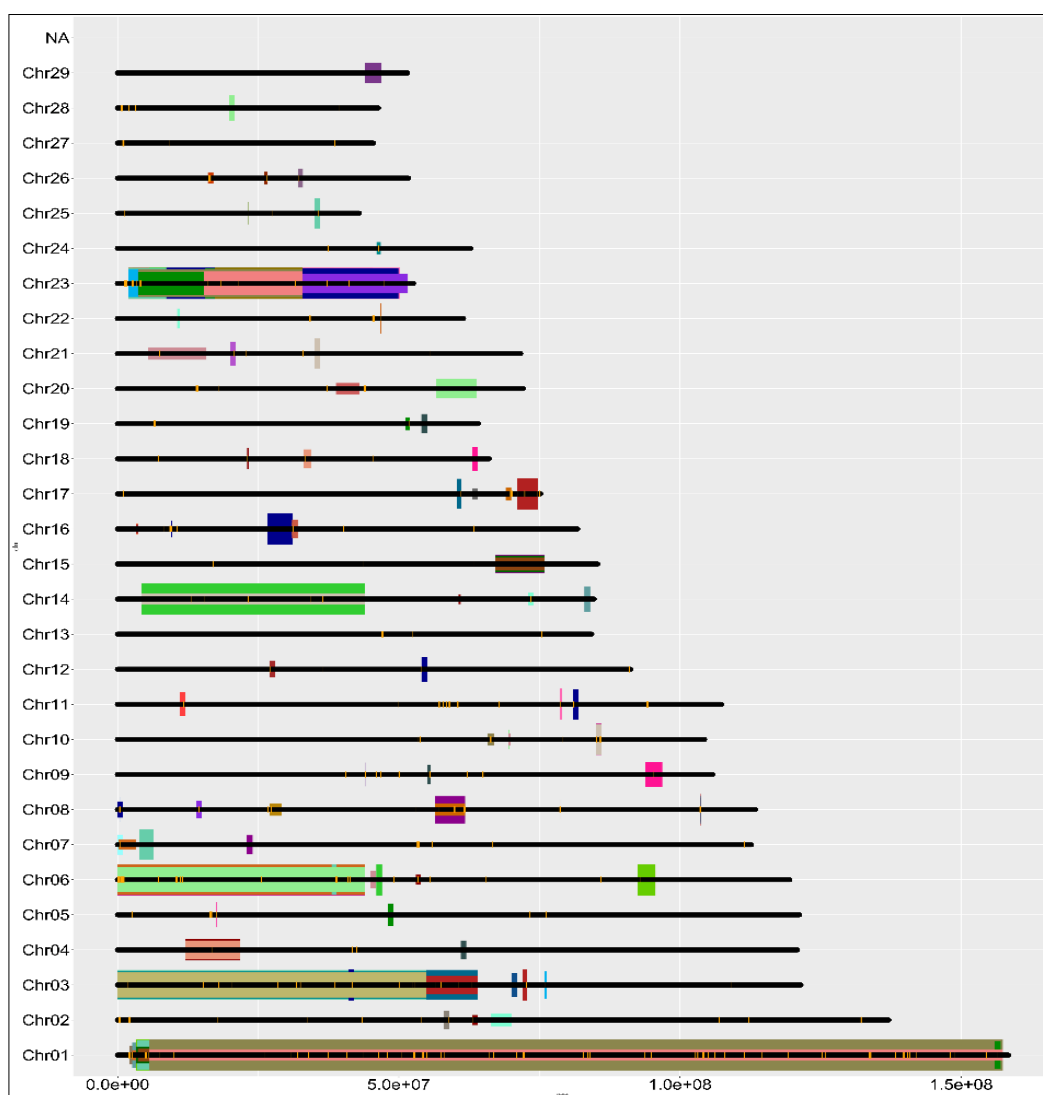

**Fig S7:**

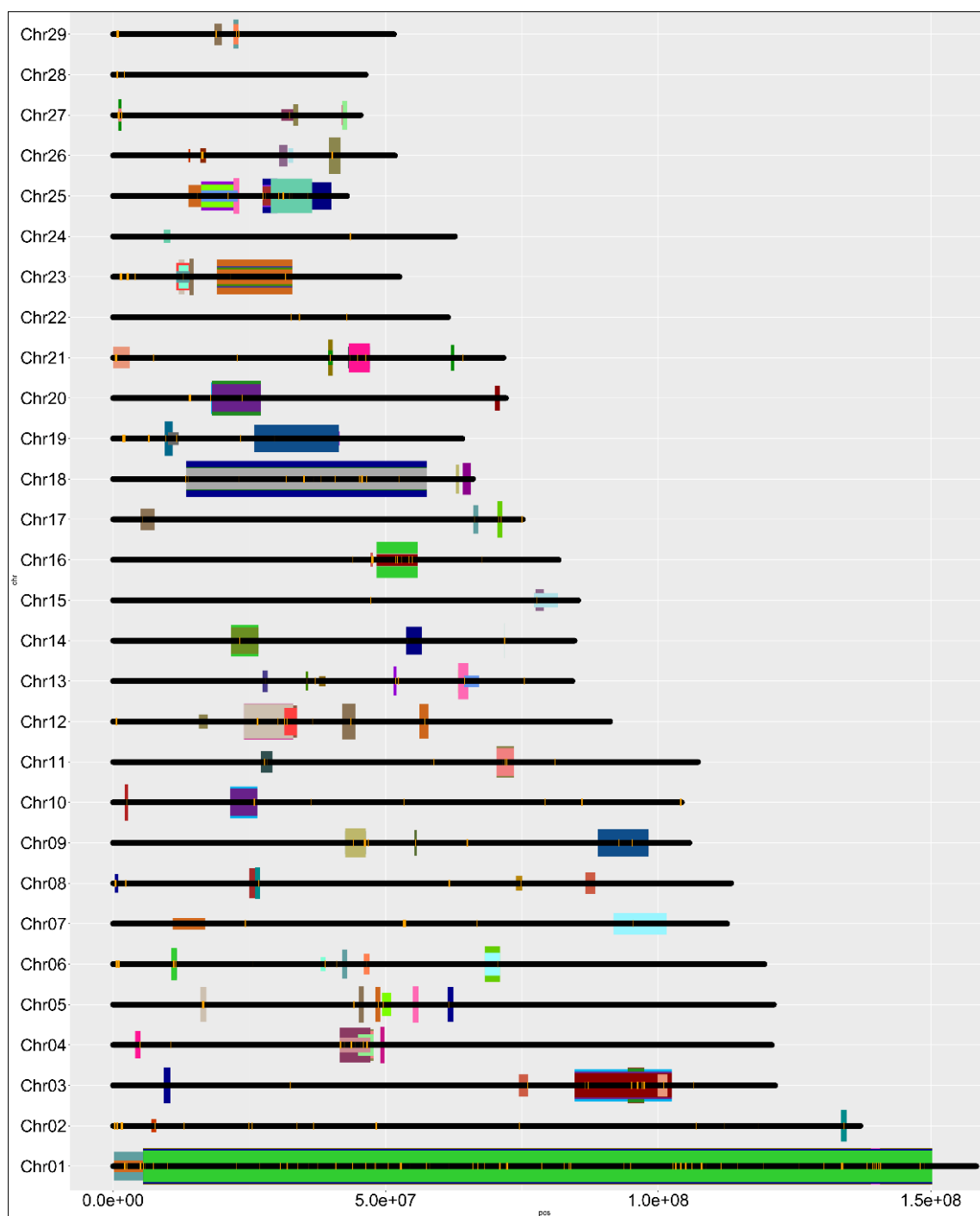

Fig S8:

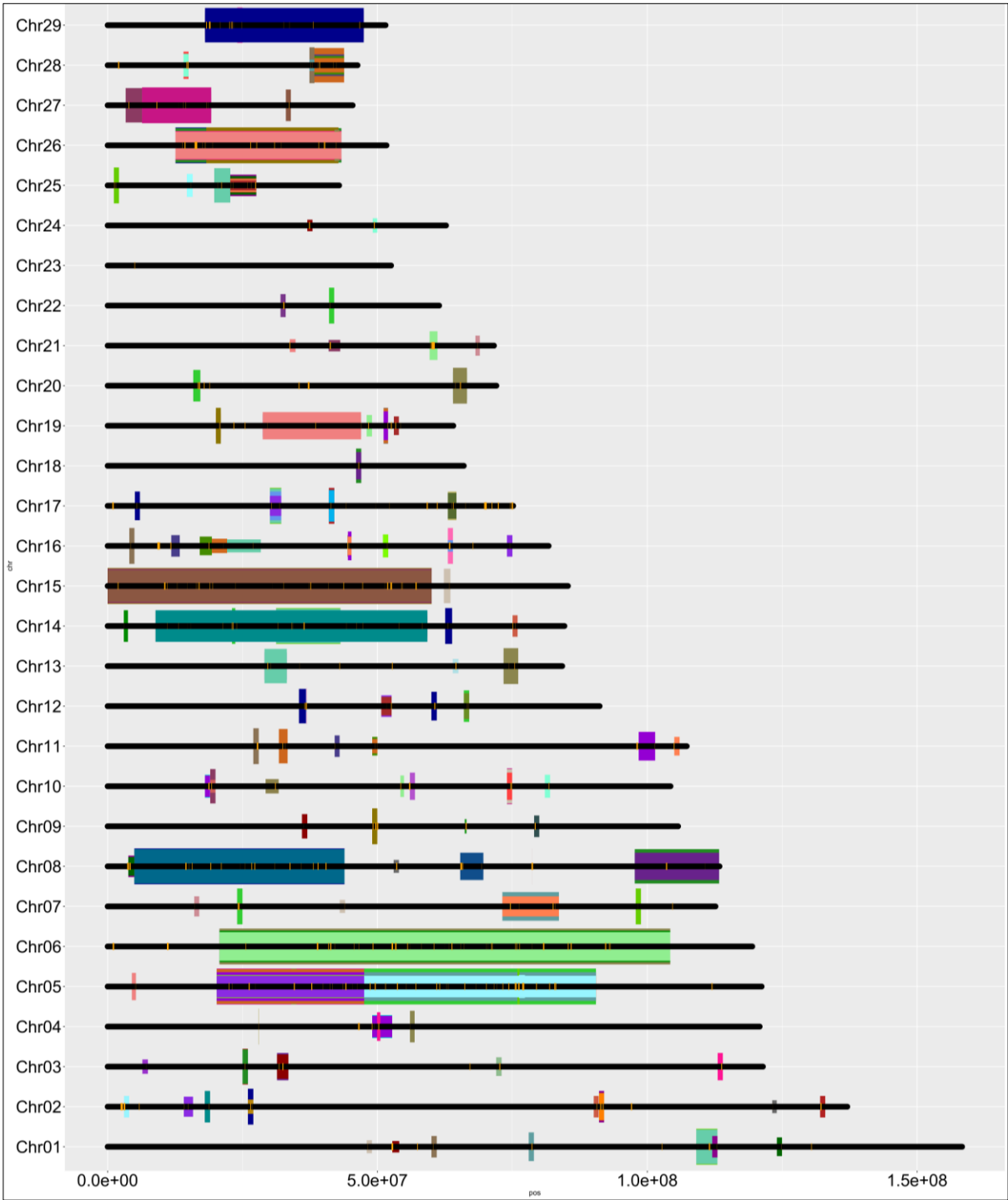

**Fig S9:**

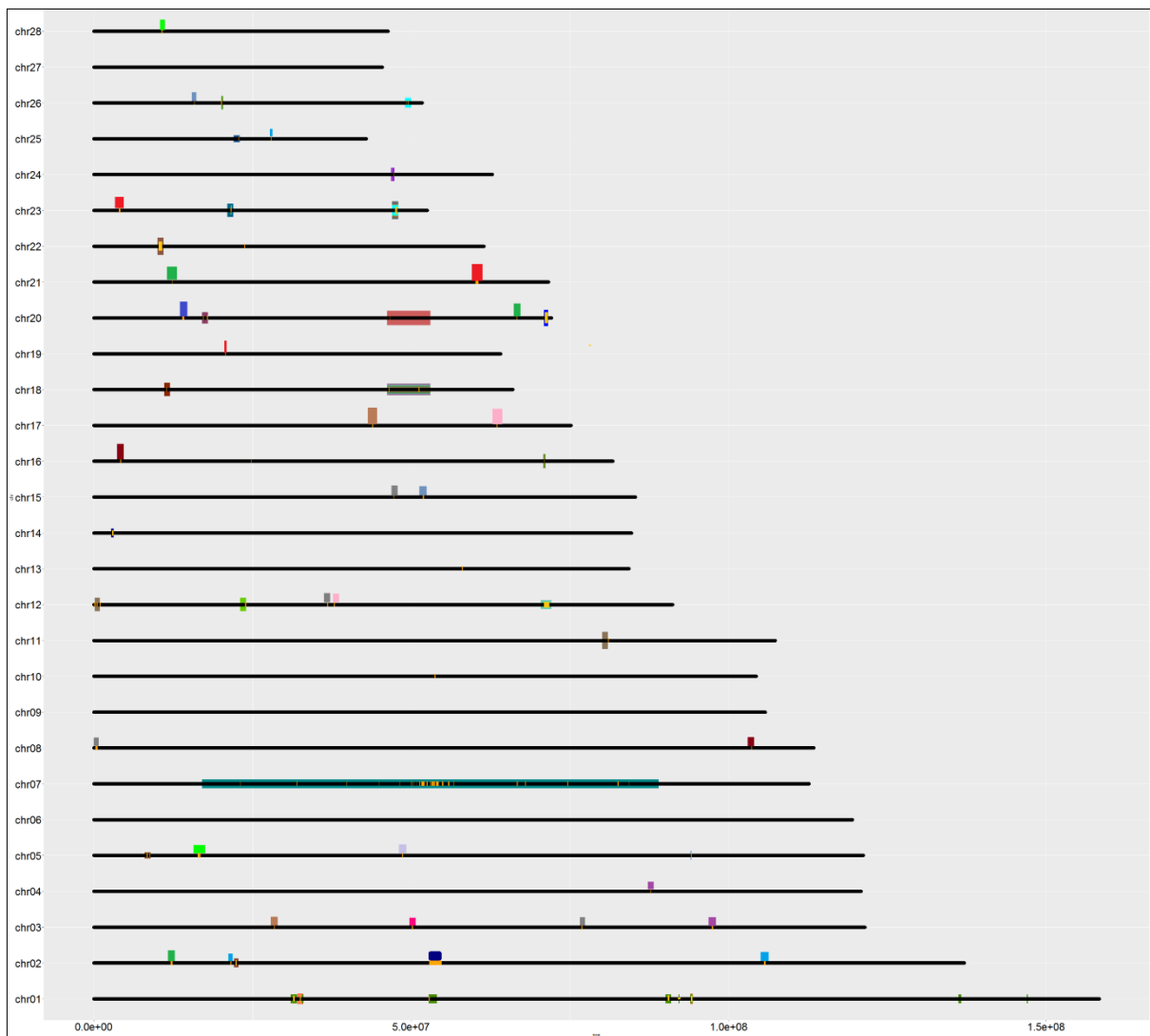

Fig S10:

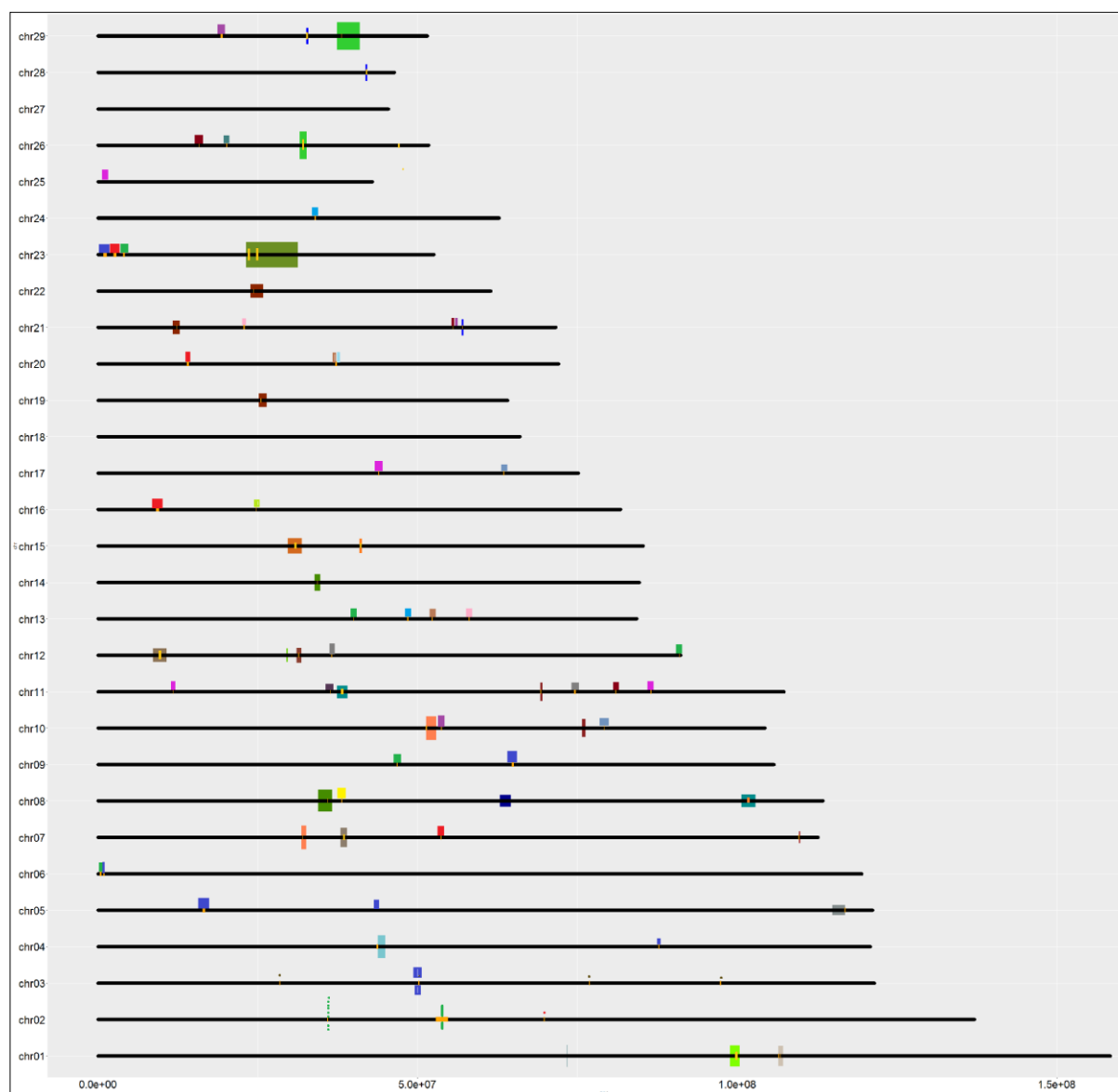

Supplement: txab033_suppl_Supplementary_Figures [file txab033_suppl_supplementary_figures.pdf]
