# Supplementary material for: High-throughput genotype-based population structure analysis of selected buffalo breeds
Source: Transl Anim Sci. 2021 May 7;5(2):txab033. doi: 10.1093/tas/txab033 (PMC8103726; doi:10.1093/tas/txab033)
Supplement: txab033_suppl_Supplementary_Tables [file txab033_suppl_supplementary_tables.docx]

**Supporting information**

**S1 Table: Number of SNPs processed during the quality control in the buffalo genotyped data processing**

| **Sr. No** | **Criteria** | **Number of SNPs** |
| --- | --- | --- |
| I | Total SNPs | **89,988** |
| II | SNPs passing filter criteria | **75,704** |
| III | SNPs discarded | **14,284** |
| 1. | Unknown chromosome-specific position | 48 |
| 2. | Chromosome X | 6,823 |
| 3. | HWE (p<0.00001) | 528 |
| 4. | Missing genotype rate less than 0.1 | 6,885 |

S2 Table: Mean pairwise F_ST_ values among various buffalo breeds with 95% of significance level (p<0.05)

(BBN: Banni, BJF: Jaffarabadi, BMR: Murrah, BNR: Nili-Ravi, BMS: Mehsana, BPN: Pandharpuri, BST: Surti)

| **Breed** | **BMR** | **BNR** | **BMS** | **BJF** | **BBN** | **BPN** | **BST** |
| --- | --- | --- | --- | --- | --- | --- | --- |
| **BMR** | 0 |  |  |  |  |  |  |
| **BNR** | 0.00221 | 0 |  |  |  |  |  |
| **BMS** | 0.00402 | 0.00599 | 0 |  |  |  |  |
| **BJF** | 0.00947 | 0.01209 | 0.01794 | 0 |  |  |  |
| **BBN** | 0.02143 | 0.00790 | 0.00442 | 0.01322 | 0 |  |  |
| **BPN** | 0.01833 | 0.02330 | 0.02188 | 0.02156 | 0.02650 | 0 |  |
| **BST** | 0.02143 | 0.02430 | 0.01794 | 0.02122 | 0.02650 | 0.03097 | 0 |

S3 Table: Chromosome-wise distribution of LD-blocks, markers and QTLs for respective Traits

| Chromosome | Total Number of Markers | | | Milk Traits | | | Meat & Carcass Trait | | | Reproduction Trait | | | Production Trait | | | Exterior Trait | | | Health Trait | | |
| --- | --- | --- | --- | --- | --- | --- | --- | --- | --- | --- | --- | --- | --- | --- | --- | --- | --- | --- | --- | --- | --- |
|  | Blocks | Markers | Density | Blocks | Markers | QTLs | Blocks | Markers | QTLs | Blocks | Markers | QTLs | Blocks | Markers | QTLs | Blocks | Markers | QTLs | Blocks | Markers | QTLs |
| 1 | 99 | 345 | 0.00022 | 89 | 306 | 137 | 12 | 41 | 10 | 93 | 322 | 19 | 98 | 339 | 139 | 5 | 19 | 13 | 7 | 24 | 6 |
| 2 | 87 | 319 | 0.00023 | 30 | 110 | 57 | 14 | 59 | 15 | 16 | 71 | 40 | 14 | 55 | 22 | 4 | 16 | 20 | 7 | 29 | 8 |
| 3 | 58 | 189 | 0.00016 | 8 | 31 | 13 | 7 | 25 | 9 | 13 | 49 | 16 | 31 | 103 | 10 | 2 | 8 | 4 | 3 | 13 | 3 |
| 4 | 58 | 200 | 0.00017 | 7 | 31 | 12 | 6 | 18 | 5 | 13 | 50 | 7 | 7 | 26 | 7 | 3 | 15 | 2 | 1 | 4 | 1 |
| 5 | 63 | 235 | 0.00019 | 14 | 54 | 32 | 47 | 162 | 20 | 10 | 34 | 21 | 7 | 32 | 13 | 2 | 9 | 12 | 5 | 20 | 5 |
| 6 | 43 | 160 | 0.00013 | 20 | 77 | 47 | 35 | 125 | 39 | 11 | 42 | 35 | 21 | 90 | 79 | 12 | 46 | 35 | 2 | 8 | 2 |
| 7 | 44 | 164 | 0.00015 | 15 | 69 | 23 | 9 | 28 | 9 | 7 | 33 | 9 | 8 | 43 | 13 | 3 | 21 | 7 | 29 | 121 | 2 |
| 8 | 52 | 195 | 0.00017 | 16 | 60 | 31 | 28 | 108 | 10 | 7 | 30 | 8 | 12 | 56 | 21 | 2 | 4 | 2 | 3 | 16 | 3 |
| 9 | 39 | 157 | 0.00015 | 9 | 45 | 17 | 7 | 30 | 4 | 8 | 41 | 12 | 9 | 44 | 16 | 2 | 10 | 14 | 1 | 5 | 2 |
| 10 | 36 | 142 | 0.00014 | 8 | 33 | 20 | 8 | 35 | 14 | 8 | 31 | 12 | 11 | 45 | 15 | 4 | 18 | 18 | 2 | 9 | 3 |
| 11 | 54 | 190 | 0.00018 | 21 | 85 | 76 | 9 | 31 | 9 | 9 | 38 | 5 | 13 | 64 | 26 | 5 | 19 | 12 | 2 | 7 | 2 |
| 12 | 37 | 139 | 0.00015 | 9 | 39 | 22 | 8 | 26 | 6 | 11 | 46 | 12 | 4 | 14 | 6 | 4 | 15 | 14 | 6 | 28 | 6 |
| 13 | 38 | 127 | 0.00015 | 18 | 72 | 118 | 8 | 29 | 6 | 10 | 36 | 20 | 4 | 20 | 14 | 3 | 11 | 18 | 1 | 2 | 1 |
| 14 | 31 | 91 | 0.00011 | 11 | 37 | 20 | 21 | 53 | 10 | 5 | 17 | 4 | 16 | 43 | 9 | 2 | 5 | 1 | 1 | 2 | 1 |
| 15 | 33 | 99 | 0.00012 | 7 | 17 | 10 | 28 | 88 | 9 | 2 | 6 | 5 | 6 | 18 | 7 | 2 | 4 | 2 | 2 | 10 | 2 |
| 16 | 44 | 157 | 0.00019 | 14 | 51 | 17 | 14 | 49 | 15 | 12 | 41 | 9 | 13 | 55 | 17 | 2 | 16 | 9 | 4 | 15 | 4 |
| 17 | 30 | 115 | 0.00015 | 18 | 51 | 69 | 21 | 88 | 9 | 5 | 25 | 5 | 8 | 57 | 8 | 0 | 0 | 0 | 1 | 5 | 1 |
| 18 | 24 | 73 | 0.00011 | 5 | 16 | 9 | 1 | 4 | 2 | 18 | 58 | 5 | 5 | 15 | 6 | 0 | 0 | 0 | 4 | 10 | 5 |
| 19 | 31 | 141 | 0.00022 | 13 | 55 | 122 | 11 | 38 | 32 | 7 | 36 | 12 | 3 | 15 | 5 | 3 | 6 | 4 | 2 | 14 | 2 |
| 20 | 23 | 80 | 0.00011 | 13 | 51 | 76 | 8 | 33 | 9 | 5 | 23 | 6 | 7 | 28 | 19 | 2 | 14 | 25 | 8 | 32 | 5 |
| 21 | 36 | 142 | 0.00020 | 6 | 23 | 7 | 6 | 30 | 5 | 11 | 43 | 16 | 6 | 22 | 11 | 3 | 21 | 12 | 3 | 18 | 6 |
| 22 | 26 | 98 | 0.00016 | 15 | 54 | 6 | 2 | 9 | 2 | 3 | 18 | 5 | 4 | 24 | 8 | 3 | 13 | 14 | 3 | 13 | 3 |
| 23 | 22 | 82 | 0.00016 | 18 | 66 | 24 | 1 | 4 | 1 | 9 | 37 | 14 | 19 | 68 | 51 | 4 | 21 | 25 | 4 | 12 | 5 |
| 24 | 27 | 80 | 0.00013 | 7 | 21 | 3 | 5 | 9 | 2 | 2 | 7 | 2 | 2 | 9 | 2 | 2 | 9 | 2 | 0 | 0 | 2 |
| 25 | 29 | 80 | 0.00019 | 8 | 24 | 76 | 12 | 31 | 4 | 13 | 41 | 11 | 5 | 14 | 5 | 2 | 6 | 3 | 2 | 5 | 0 |
| 26 | 16 | 57 | 0.00011 | 13 | 49 | 162 | 14 | 51 | 5 | 6 | 24 | 7 | 3 | 15 | 3 | 2 | 4 | 2 | 1 | 4 | 1 |
| 27 | 23 | 77 | 0.00017 | 12 | 41 | 42 | 8 | 24 | 3 | 5 | 19 | 7 | 4 | 15 | 6 | 0 | 0 | 0 | 0 | 0 | 0 |
| 28 | 19 | 73 | 0.00016 | 7 | 33 | 9 | 9 | 30 | 4 | 2 | 10 | 7 | 5 | 22 | 6 | 1 | 2 | 1 | 0 | 0 | 0 |
| 29 | 22 | 83 | 0.00016 | 5 | 23 | 17 | 9 | 27 | 3 | 5 | 25 | 5 | 0 | 0 | 1 | 2 | 6 | 2 | 0 | 0 | 0 |
| Total | 1144 | 4090 | 0.00462 | 436 | 1624 | 1274 | 368 | 1285 | 271 | 326 | 1253 | 336 | 345 | 1351 | 545 | 81 | 338 | 273 | 104 | 426 | 81 |

**Fig S1: Sampling across different geographical locations of India.**

**Fig S2: Breed-wise average alternate allele frequency distribution.**

(BBN: Banni, BJF: Jaffarabadi, BMR: Murrah, BNR: Nili-Ravi, BMS: Mehsana, BPN: Pandharpuri, BST: Surti)

Fig S3: Phylogenetic Tree of breed differentiation based on allele frequency difference among breeds (BBN: Banni, BJF: Jaffarabadi, BMR: Murrah, BNR: Nili-Ravi, BMS: Mehsana, BPN: Pandharpuri, BST: Surti)

Fig S4: Marginal likelihood plot describing the maximum probability of population differentiation at K=4 level.

Fig S5: Concordance of LD blocks with QTLs of Milk production traits on A) chromosome one and on B) chromosomes number two to 29

Vertical axis shows the chromosome number, horizontal axis shows the base pair position, thick middle black bar shows physical length of chromosome, thin orange colored bars over black bars shows LD blocks and the colored segments reflects the physical length of QTLs.

Fig S6: Concordance of LD blocks with QTLs of Production traits.

Vertical axis shows the chromosome number, horizontal axis shows the base pair position, thick middle black bar shows physical length of chromosome, thin orange colored bars over black bars shows LD blocks and the colored segments reflects the physical length of QTLs

Fig S7: Concordance of LD blocks with QTLs of Reproduction traits.

Vertical axis shows the chromosome number, horizontal axis shows the base pair position, thick middle black bar shows physical length of chromosome, thin orange colored bars over black bars shows LD blocks and the colored segments reflects the physical length of QTLs

Fig S8: Concordance of LD blocks with QTLs of Meat and carcass traits. Vertical axis shows the chromosome number, horizontal axis shows the base pair position, thick middle black bar shows physical length of chromosome, thin orange colored bars over black bars shows LD blocks and the colored segments reflects the physical length of QTLs

Fig S9: Concordance of LD blocks with QTLs of Health trait.

Vertical axis shows the chromosome number, horizontal axis shows the base pair position, thick middle black bar shows physical length of chromosome, thin orange colored bars over black bars shows LD blocks and the colored segments reflects the physical length of QTLs

Fig S10: Concordance of LD blocks with QTLs of Exterior traits.

Vertical axis shows the chromosome number, horizontal axis shows the base pair position, thick middle black bar shows physical length of chromosome, thin orange colored bars over black bars shows LD blocks and the colored segments reflects the physical length of QTLs.
